# Two New Thymol Derivatives from the Roots of *Ageratina adenophora*

**DOI:** 10.3390/molecules22040592

**Published:** 2017-04-08

**Authors:** Li-Mei Dong, Mei Zhang, Qiao-Lin Xu, Qiang Zhang, Bi Luo, Qing-Wen Luo, Wen-Bin Liu, Jian-Wen Tan

**Affiliations:** 1 Guangdong Provincial Key Laboratory of Applied Botany, South China Botanical Garden, Chinese Academy of Sciences, Guangzhou 510650, China; donglimei1990@163.com (L.-M.D.); mzhang309@163.com (M.Z.); zqiang55@126.com (Q.Z.); bluo023@163.com (B.L.); luoyaa@foxmail.com (Q.-W.L); liuwenbin15@mails.ucas.ac.cn (W.-B.L.); 2 State Key Laboratory for Conservation and Utilization of Subtropical Agro-Bioresources/Guangdong Key Laboratory for Innovative Development and Utilization of Forest Plant Germplasm, College of Forestry and Landscape Architecture, South China Agricultural University, Guangzhou 510642, China; 3 College of Life Sciences, University of Chinese Academy of Sciences, Beijing 100049, China; 4 Guangdong Provincial Key Laboratory of Bio-Control for the Forest Disease and Pest, Guangdong Academy of Forestry, Guangzhou 510520, China; qlxu@sinogaf.cn

**Keywords:** *Ageratina adenophora*, thymol derivatives, monoterpenes, antibacterial, cytotoxicity

## Abstract

Two new thymol derivatives, 7,9-diisobutyryloxy-8-ethoxythymol (**1**) and 7-acetoxy-8-methoxy-9-isobutyryloxythymol (**2**), were isolated from fresh roots of *Ageratina adenophora*, together with four known compounds, 7,9-di-isobutyryloxy-8-methoxythymol (**3**), 9-oxoageraphorone (**4**), (−)-isochaminic acid (**5**) and (1α,6α)-10-hydroxycar-3-ene-2-one (**6**). Their structures were established on the basis of detailed spectroscopic analysis, and they were all isolated from the roots of *A. adenophora* for the first time. All the compounds were tested for their in vitro antibacterial activity toward three Gram-positive and two Gram-negative bacterial strains. Thymol derivatives **1**–**3** only selectively showed slight in vitro bacteriostatic activity toward three Gram-positive bacteria. The two known carene-type monoterpenes **5** and **6** were found to show moderate in vitro antibacterial activity against all five tested bacterial strains, with MIC values from 15.6 to 62.5 μg/mL. In addition, compounds **5** and **6** were further revealed to show in vitro cytotoxicity against human tumor A549, HeLa and HepG2 cell lines, with IC_50_ values ranging from 18.36 to 41.87 μM. However, their cytotoxic activities were inferior to those of reference compound adriamycin.

## 1. Introduction

*Ageratina adenophora* (Spreng.) King and Robinson is a perennial, herbaceous invasive plant, native to Mexico and Costa Rica, which has invaded around 30 countries in tropical and subtropical zones of the world [1,2]. This plant was first introduced to Yunnan Province of China in the 1940s, and by now it has rapidly spread across a large area of southwest China, including Yunnan, Guizhou, Guangxi, Sichuan, Chongqing and Xizang provinces [3]. The rapid spread of *A. adenophora* in China has caused serious economic losses to agriculture, forestry and livestock, and damaged the ecology and environment of China’s native habitat [4,5].

**Figure 1:**
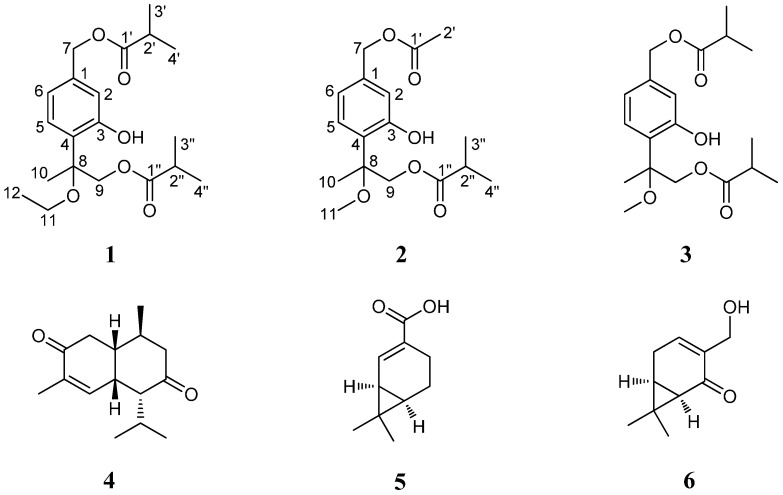
Chemical structures of compounds **1**−**6**.

*A. adenophora* is seldom attacked by microorganisms (including bacteria and fungi) and insects, suggesting that rich bioactive secondary metabolites that might be defense-related, would exist in this plant. Previous phytochemical studies have revealed structurally diverse chemicals including (mono-, sesqui-, di-, and tri-) terpenoids, phenylpropanoids, flavonoids, coumarins, sterols and alkaloids were reported from this species [6,7,8], some of which were shown to possess allelopathic [9,10], phytotoxic [11] and antifeedant [12] activities. Our recent study also revealed some bioactive natural products, including bioactive quinic acid derivatives and monoterpenes from the aerial parts of *A. adenophora* and some phenolic compounds with allelopathic potential from the roots of this species [13,14,15]. In continuation of our work on searching for bioactive natural compounds of *A. adenophora*, a further study on the roots of this invasive plant was conducted, which led to the isolation of two new thymol derivatives (**1** and **2**) along with four known compounds (**3**–**6**) (Figure 1). Herein, we report the isolation and structural elucidation of these compounds, as well as their antibacterial and cytotoxic activities.

## 2. Results and Discussion

The EtOH extract of the fresh roots of *A. adenophora* was initially partitioned between water and EtOAc. The EtOAc soluble fraction was then subjected to silica gel column chromatography (CC) followed by CCs of silica gel, reverse phase silica gel and Sephadex LH-20, to yield new thymols **1** and **2** along with other four known metabolites, 7,9-di-isobutyryloxy-8-methoxythymol (**3**) [16], 9-oxoageraphorone (**4**) [17], (−)-isochaminic acid (**5**) [18] and (1α,6α)-10-hydroxy-3-carene-2-one (**6**) [14]. The structures of the known compounds were determined by interpretation of their spectroscopic data, as well as by comparison with reported values.

**Figure 2:**
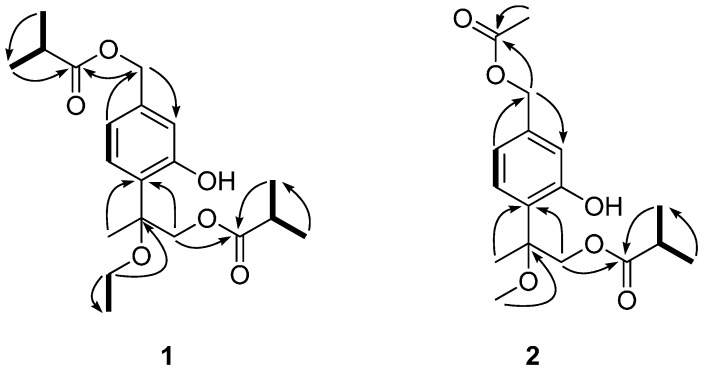
Selected HMBC (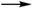) and COSY (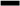) correlations of compounds **1** and **2**.

Compound **1** was isolated as a yellowish oil. Its molecular formula C_20_H_30_O_6_ was determined by HR-ESI-MS at *m/z* 389.1942 [M + Na]^+^ (calcd. for C_20_H_30_O_6_Na, 389.1940) (see the supplementary), corresponding to 6 degrees of unsaturation. The IR spectrum displayed absorptions at 3434 and 1737 cm^−1^ indicative of the existence of hydroxyl and carbonyl groups. In the ^1^H-NMR spectrum, signals for an oxymethylene at δ_H_ 5.05 (s, 2H), one tertiary methyl at δ_H_ 1.67 (s, 3H), four secondary methyls at δ_H_ 1.20 (d, 6H) and 1.13 (d, 6H), a primary methyl at δ_H_ 1.25 (t, 3H), and a 1,3,4-trisubstituted phenyl group were readily recognized. In the ^13^C-NMR and DEPT spectra (Table 1), 20 carbons including six methyls, three oxygenated methylenes, two methines, one quaternary carbon, two carboxyl carbons and six aromatic carbons (3 × C and 3 × CH) were displayed. These above data and literature precedents supported **1** to be a thymol derivative with one ethoxy group and two isobutyryloxy groups in the molecule [16,19]. Careful comparison showed that the ^1^H- and ^13^C-NMR spectroscopic data (see Table 1) of **1** were very close to those of 7,9-di-isobutyryloxy-8-methoxythymol [16], a literature reported thymol derivative which was also obtained in the present study as compound **3**, except that the resonances for the methoxy group in **3** were replaced by signals (δ_H_ 3.54 (H-11a), 3.39 (H-11b), 1.25 (H_3_-12); δ_C_ 59.1 (C-11), 15.4 (C-12)) for an ethoxy group in **1**. These findings suggested **1** to be a thymol derivative close to **3**, with only difference of the methoxy group at C-8 in **3** being replaced by an ethoxy group (Figure 1). This proposed structure was well supported by 2D NMR analyses including ^1^H-^1^H COSY and HMBC experiments (Figure 2). In the ^1^H-^1^H COSY spectrum, signals correlated to the four H-atom coupling systems, i.e., C-5 through C-6, C-11 through C-12, C-2′ through C-4′ and C-2″ through C-4″ were all exhibited. The observation of ^1^H-^13^C long-range correlation signals in the HMBC spectrum of H-9 and H-3″(4″) with C-1″ (δ_C_ 176.9) (Figure 2) evidenced the location of an isobutyryloxy group at C-9 (δ_C_ 68.3). The location of the ethoxy group at C-8 was assigned by significant HMBC correlations of H-11 with C-8 (δ_C_ 80.8). The ester bond linkage of C-7 with the other isobutyryloxy group at C-1′ was supported by the observation of HMBC correlations of H-7 (δ_H_ 5.04) and H-3′(4′) (δ_H_ 1.20) with C-1′ (δ_C_ 176.6). Therefore, compound **1** was determined as 7,9-diisobutyryloxy-8-ethoxythymol.

Compound **2** was also obtained as a yellowish oil. Its molecular formula C_17_H_24_O_6_ was deduced from the HR-ESI-MS *m/z* 347.1479 [M + Na]^+^ (calcd. for C_17_H_24_O_6_Na, 347.1471). Its spectral features and physicochemical properties suggested **2** to be also a thymol derivative. Careful comparison indicated that the ^1^H and ^13^C (DEPT) NMR data (Table 1) of **2** were very similar to those of **3**, except that the resonances for the isobutyryloxy group that located at C-7 in **3** were replaced by the signals (δ_H_ 2.10 (3H, s, H_3_-2′); δ_C_ 170.8 (C-1′), 20.9 (C-2′)) for an acetoxy group in **2** (Figure 1). This deduction was supported by its molecular formula and the observation of significant ^1^H-^13^C long-range correlation signals in the HMBC spectrum of H-7 (δ_H_ 5.03) and H-2′ (δ_H_ 2.10) with C-1′ (δ_C_ 170.8) (Figure 2). By further detailed analysis of 2D NMR (^1^H-^1^H COSY, HSQC, and HMBC) spectra (Figure 2), the ^1^H- and ^13^C spectroscopic NMR data were unambiguously assigned as shown in Table 1, and they were fully supported by the structure of **2** as shown in Figure 1. Accordingly, the structure of **2** was thus determined as 7-acetoxy-8-methoxy-9-isobutyryloxythymol.

Among these isolated compounds, **1**–**3** were thymol derivatives. Thymols, first found in *Thymus* plants [20], were recently discovered to be in rich supply in *Eupatorium* species [19,21,22]. Due to the thymol derivatives usually being oily like compounds which were difficult to prepare single crystals for X-ray analysis, it is generally difficult to determine the stereochemistry of thymols at the stereogenic center C-8 [19,23]. Just like the thymol precedents in the literature, the stereochemistry of thymols **1**–**3** at the stereogenic center C-8 were also still an open question. Compounds **5** and **6** were two carene-type monoterpenes with potential anti-fungal activities that we have recently obtained from the aerial parts of *A. adenophora* [14]. To the best of our knowledge, this is the first time these compounds have been obtained from the roots of this plant.

These six compounds were tested for their in vitro antibacterial activities against five bacterial strains, including three Gram-positive bacteria of *Staphylococcus aureus*, *Bacillus thuringiensis* and *Bacillus subtilis*, and two Gram-negative bacteria of *Escherichia coli* and *Shigella dysenteria*. The experimental results obtained from the bioassay (Table 2) showed that the two carene-type monoterpenes **5** and **6** were moderately active toward the five bacterial strains with MIC values from 15.6 to 62.5 μg/mL. The thymol compounds **1**–**3** only selectively showed weak bacteriastatic activity toward the three Gram-positive bacterial strains, and no antibacterial activity was detected for compound **4** in this bioactive assay.

Compounds **1**–**6** were further evaluated for their in vitro cytotoxicity against three human cancer cell lines (A549, HeLa and HepG2) using a MTT method as described. The resulting IC_50_ values are displayed in Table 3, as compared to adriamycin as positive control. Compounds **5** and **6** showed moderate cytotoxicity against all the three tested cancer cell lines, with IC_50_ values ranging from 18.36 to 41.87 μM. No obvious activity was detected for the other four isolates (**1**–**4**).

*A. adenophora* is a well-known invasive plant which has spread rapidly in the southwest part of China. To date, phytochemical investigations have revealed diverse chemicals from this plant species. However, those studies were mainly focused on the aerial parts, and seldom concentrated on the roots. Our recent study revealed a series of phenolic compounds with allelopathic potential from the roots of this plant [15]. The present study further indicated that the roots of *A. adenophora* are rich in potential bioactive compounds worthy of further investigation.

## 3. Materials and Methods

### 3.1. General Information

Optical rotations were obtained on a Perkin-Elmer 341 polarimeter (Perkin-Elmer, Waltham, MA, USA) with MeOH as solvent. UV spectra were recorded in MeOH on a Perkin-Elmer Lambda 35 UV-vis spectrophotometer. IR spectra (KBr) were recorded on a Bruker Tensor 27 spectrophotometer in cm^−1^. ^1^H (600 MHz and 400 MHz), ^13^C (150 MHz and 100 MHz), and 2D NMR spectra were recorded in CDCl_3_ and CD_3_OD on a Bruker DRX-400 instrument and a Bruker AVANCE 600 instrument with TMS as an internal standard. HR-ESI-MS data were obtained on a Water Q-TOF Premier mass spectrometer and HR-EI-MS data were obtained on a Finigan MAT 95XP mass spectrometer. ESIMS were collected on an MDS SCIEX API 2000 LC/MS/MS instrument. Preparative HPLC was conducted using a CXTH P3000 HPLC pump and a UV3000 UV-Vis Detector with a Fuji-C18 column (10 µm–100A, ChuangXinTongHeng Science And Technology Co., Ltd., Beijing, China). For column chromatography (CC), silica gel (200–300 mesh, Qingdao Marine Chemical Inc.), YMC ODS-A (50 μm, YMC Co. Ltd., Kyoto, Japan) were used, and Sephadex LH-20 (Pharmacia Fine Chemical Co. Ltd., Uppsala, Sweden) were used. Analytical grade petroleum ether (b.p. 60–90 °C), methanol, ethyl acetate, chloroform, acetone were purchased from Tianjin Fuyu Fine Chemical Industry Co. (Tianjin, China); HPLC grade methanol was purchased from J&K Chemical Ltd. (Beijing, China); Fractions were monitored by pre-coated HSGF_254_ TLC Yantai Jiangyou Silica Gel Co. Ltd. (Yantai, China), and spot detection was performed under fluorescent light (λ = 254 nm), and then spraying 10% H_2_SO_4_ in ethanol, followed by heating. RPMI-1640 medium and fetal calf serum were purchased from Gibco BRL (Gaithersburg, MD, USA). Kanamycin sulfate, Resazurin, Cefradine, CDCl_3_, 3-(4,5-dimethylthiazol-2-yl)-2,5-diphenyltetrazolium bromide (MTT) was purchased from Sigma Chemical Co. (Sigma-Aldrich, St. Louis, MO, USA). Adriamycin was obtained from Pfizer Italia SRL (Roma, Italy). *p*-Nitrophenyl-α-d-glucopyranoside (PNPG) were obtained from Tokyo Chemical Industry Co., Ltd. (Tokyo, Japan).

### 3.2. Plant Materials

The root material of *A. adenophora* was collected in a suburb of Kunming, Yunnan province, P. R. China, in September 2010, identified by one of the authors (J.-W.T.). A voucher specimen (No. 20100902) was deposited at the Laboratory of Phytochemistry at the South China Botanical Garden, Chinese Academy of Sciences.

### 3.3. Extraction and Isolation

The fresh root material (14.5 kg) of *A. adenophora* were cut into pieces and extracted three times with 95% EtOH (3 × 20 L) at room temperature. After removal of the EtOH in vacuo, the viscous concentrate was suspended in 10% ethanol in H_2_O (2.5 L) and extracted with ethyl acetate (EtOAc) (3 × 2.5 L). The oily EtOAc extract (76 g) was then applied on silica gel (500 g) column chromatography and eluted with 2L petroleum ether/CHCl_3_ (1:1, *v*/*v*) to yield an olily fraction (22.1 g) which was further subjected to silica gel column chromatography using a gradient of petroleum ether/CHCl_3_ (90:10–0:100, *v*/*v*) and CHCl_3_–MeOH (100:0–80:20, *v*/*v*) to give sub-fractions F_1_–F_7_. The fraction F_2_ (4.0 g), obtained from the elution of petroleum ether/CHCl_3_ of 1:1, was separated by RP-C_18_ MPLC eluted with MeOH/H_2_O (from 4:6 to 7:3, *v*/*v*) and further purified by Sephadex LH-20 column chromatography with elution solvent of MeOH to give compound **5** (2.6 mg). The fraction F_4_ (4.7 g), obtained from the elution of CHCl_3_/MeOH of 95:5, was applied on silica gel (200–300 mesh) column chromatography and eluted with a gradient of *n*-hexane/ethyl acetate to afford fractions F_4-1_–F_4-8_ on the basis of thin layer chromatography (TLC) profiles. Fraction F_4__-3_ (1.3 g), obtained from the elution of *n*-hexane/ethyl acetate of 8.5:1.5, was separated by silica gel column chromatography eluted with petroleum ether/Me_2_CO (from 9:1 to 7:3, *v*/*v*) to give subfractions F_4-3-1_–F_4-3-5_, of which subfraction F_4-3-4_ (0.4 g) was further purified by RP-C_18_ HPLC eluted with 70% MeOH in H_2_O to afford compound **1** (t_R_ = 37.2 min, 2.8 mg). Fraction F_4-__4_ (1.1 g), obtained from the elution of *n*-hexane/ethyl acetate of 7:3, was separated by RP-C_18_ HPLC eluted with a MeOH/H_2_O gradient (from 50:50 to 80:20, *v*/*v*) to provide pure compound **4** (t_R_ = 68.1 min, 5.6 mg). Fraction F_4-6_ (0.7 g) was divided into subfractions F_4-6-1_–F_4-6-4_, of which subfraction F_4-6-4_ (39.2 mg) was purified repeatedly by Sephadex LH-20 columns eluted with CHCl_3_/MeOH (20:80, *v*/*v*) to afford compound **2** (3.0 mg). Fraction F_4-6-3_ was separated by RP-C_18_ MPLC eluted with MeOH/H_2_O (from 50:50–100:0, *v*/*v*) to attain subfractions F_4-6-3-1_–F_4-6-3-4_. Subfraction F_4-6-3-1_ was then further purified by RP-C_18_ MPLC eluted with MeOH/H_2_O (from 50:50–100:0, *v*/*v*) to attain pure compound **6** (t_R_ = 13.5 min, 2.4 mg), and subfraction F_4-6-3-3_ was purified by Sephadex LH-20 column eluted with acetone to afford compound **3** (4.7 mg).

*7,9-Diisobutyryloxy-8-ethoxythymol* (**1**). yellowish oil; [α]${}_{D}^{20}$ −5.0 (*c* 0.06, CHCl_3_); IR (KBr) ν_max_ 3434, 1737, 1265, 1187, 1155 cm^−1^; UV (MeOH) λ_max_ (log *ε*) nm: 205 (4.16), 279 (3.31); ESI-MS *m/z* 755 [2M + Na]^+^, 389 [M + Na]^+^; HR-ESI-MS *m/z* 389.1942 [M + Na]^+^ (calcd. for C_20_H_30_O_6_Na, 389.1940); For ^1^H (CDCl_3_, 400 MHz) and ^13^C NMR (CDCl_3_, 100 MHz) data, see Table 1.

*7-Acetoxy-8-methoxy-9-isobutyryloxythymol* (**2**). yellowish oil; [α]${}_{D}^{20}$ +3.8 (*c* 0.87, CHCl_3_); IR (KBr) ν_max_ 3318, 1739, 1629, 1577 cm^−1^; UV (MeOH) λ_max_ (log *ε*) nm: 204 (4.27), 219 (3.80), 279 (3.31); ESI-MS *m*/*z* 687 [2M + K]^+^, 671 [2M + Na]^+^, 363 [M + K]^+^, 347 [M + Na]^+^, 683 [2M + Cl]^−^, 359 [M + Cl]^−^, 323 [M–H]^−^; HR-ESI-MS *m/z* 347.1479 [M + Na]^+^ (calcd. for C_17_H_24_O_6_Na, 347.1471). For ^1^H (CDCl_3_, 600 MHz) and ^13^C NMR (CDCl_3_, 150 MHz) data, see Table 1.

### 3.4. Antibacterial Assay

The antibacterial activities of **1**–**6** were tested by using a microdilution method as we described previously [24]. Three Gram-positive bacteria strains, *Staphylococcus aureus, Bacillus thuringiensis and Bacillus subtilis*, and two Gram-negative bacterial species, *Escherichia coli and Shigella dysenteria*, were used in the bioassay. In the test, indicator solution of resazurin (100 μg/mL, 100 μL) was first placed into each control wells (11th column) in 96-well microplates for the assay. Subsequently, indicator solution (100 μg/mL, 7.5 mL) was mixed with test organism (10^6^ cfu/mL, 5 mL) followed by transferring (100 μL, each) to growth control wells (12th column) and all test wells (1–10th column) in the 96-well microplates. Then, each of the sample solutions (1.0 mg/mL of test compounds in methanol, 100 μL) and positive control solution (1.0 mg/mL of kanamycin sulfate or cefradine in methanol) as well as negative control sample (pure MeOH) were applied to the wells in the 1st column of the plates. In each test microplate, the six compound samples along with positive control and negative control samples were applied. Once all controls and samples were properly applied to the 1st column of wells in the microplates, half of the homogenized content (100 μL) from these wells was then transferred parallel to the 2nd column of wells, and each subsequent column of wells was treated similarly (doubling dilution) up to the 10th column, followed by discarding the last 100 μL aliquot. Finally, the plates were incubated at 37 °C for 5–6 h until the color of growth control wells change to pink. The lowest concentration for each test compound at which color change occurred was recorded as its primary MIC value. The averages of the primary values from three individual tests were calculated and these were taken as the final MIC values for the test compounds. MIC values for test compounds were displayed in Table 2.

### 3.5. Cytotoxic Assay

Compounds **1**–**6** were evaluated for their cytotoxity against human lung adenocarcinoma (A549), human cervical carcinoma (HeLa) and human liver hepatocellular carcinoma (HepG2) cell lines. The three tumor cell lines were obtained from Kunming Institute of Zoology, Chinese Academy of Sciences. The cytotoxic activities of the tested compounds were assayed according to an MTT method by using 96 well plates [25]. In brief, the cells were cultured in RPMI-1640 medium, supplemented with 10% fetal bovine serum in a humidified atmosphere with 5% CO_2_ at 37 °C. 100 μL adherent cells at the density of 5 × 10^4^ cell/mL was seeded into each well of 96-well cell culture plates and incubated in 5% CO_2_ at 37 °C for 24 h to form a monolayer on the flat bottoms. Then, the supernatant per well was removed and subsequently added with 100 μL fresh medium and 100 μL medium containing a test compound. The plate was then incubated in 5% CO_2_ at 37 °C. After 72 h, 20 μL of 5 mg/mL MTT in DMSO was added into each well and incubated for 4 h. The supernatant per well was carefully removed and 150 μL DMSO was added. The plate was then vortex shaken for 15 min to dissolve blue formazan crystals. The optical density (OD) of each well was measured on a Genois microplate reader (Tecan GENios, Männedorf, Switzerland) at the wavelength of 570 nm. All assays were performed in triplicate and adriamycin was used as a positive control. In each experiment, each of the tumor cell lines was exposed to the test compound at concentrations 50, 25, 12.5, 6.25, 3.125, 1.5625 µg/mL. The inhibitory rate of cell growth was calculated according to the following formula: Inhibition rate (%) = (OD_control_ − OD_treated_)/OD_control_ × 100%. IC_50_ values were calculated by SPSS 16.0 statistic software. The values were based on three individual experiments and expressed as means ± standard deviation (SD).

## 4. Conclusions

Six natural products, including two new thymol derivatives **1** and **2**, were isolated from the roots of *A. adenophora*. Their structures were identified by extensive spectroscopic analysis, including NMR and HR-ESI-MS techniques. All the compounds were isolated from the roots of *A. adenophora* for the first time. Thymol compounds **1**−**3**, and monoterpenes **5** and **6** selectively showed in vitro antibacterial activity against five assayed bacterial strains. Compounds **5** and **6** were further revealed to show moderate in vitro cytotoxicity against human tumor A549, HeLa and HepG2 cell lines. The present study indicates that the roots of *A. adenophora* are also rich in potential bioactive compounds worthy of further investigation.

## Figures and Tables

**Table 1:** The ^1^H- and ^13^C-NMR data of compounds **1**, **2** and **3**.

H/C	1 ^a^		2 ^a^		3 ^b^	
	δ_H_ (Mult., *J* in Hz)	δ_C_ (DEPT)	δ_H_ (Mult., *J* in Hz)	δ_C_ (DEPT)	δ_H_ (Mult., *J* in Hz)	δ_C_ (DEPT)
1		138.2 (s)		137.9 (s)		138.3 (s)
2	6.84 (d, 1.2)	116.3 (d)	6.85 (s)	116.6 (d)	6.86 (s)	116.4 (d)
3		156.5 (s)		156.4 (s)		156.4 (s)
4		124.4 (s)		123.8 (s)		123.6 (s)
5	7.01 (d, 8.0)	127.6 (d)	7.01 (d, 8.4)	127.8 (d)	7.02 (d, 8.4)	127.9 (d)
6	6.82 (dd, 8.0, 1.2)	118.8 (d)	6.82 (d, 8.4)	119.2 (d)	6.83 (d, 8.4)	119.0 (d)
7	5.04 (s)	65.3 (t)	5.03 (s)	65.5 (t)	5.05 (s)	65.3 (t)
8		80.8 (s)		81.3 (s)		81.4 (s)
9	4.36 (d, 11.6)	68.3 (t)	4.33 (d, 12.0)	68.2 (t)	4.34 (d, 11.6)	68.3 (t)
	4.23 (d, 11.6)		4.22 (d, 12.0)		4.23 (d, 11.6)	
10	1.66 (s)	21.0 (q)	1.64 (s)	20.2 (q)	1.65 (s)	20.2 (q)
11	3.54 (m), 3.39 (m)	59.1 (t)	3.28 (s)	50.9 (q)	3.29 (s)	51.0 (q)
12	1.25 (t, 7.2)	15.4 (q)				
1′		176.6 (s)		170.8 (s)		176.6 (s)
2′	2.56 (m)	34.0 (d)	2.10 (s)	20.9 (q)	2.60 (m)	33.9 (d)
3′	1.20 (d, 6.8)	19.0 (q)			1.20 (d, 6.8)	18.8 (q)
4′	1.20 (d, 6.8)	19.0 (q)			1.20 (d, 6.8)	18.9 (q)
1“		176.9 (s)		176.5 (s)		176.9 (s)
2“	2.62 (m)	34.0 (d)	2.55 (m)	33.9 (d)	2.60 (m)	34.0 (d)
3“	1.13 (d, 6.8)	18.9 (q)	1.12 (d, 6.6)	18.8 (q)	1.12 (d, 6.8)	18.9 (q)
4“	1.13 (d, 6.8)	18.9 (q)	1.13 (d, 6.6)	18.8 (q)	1.13 (d, 6.8)	20.2 (q)

**^a^** Recorded in CDCl_3_; ^b^ Recorded in CD_3_OD.

**Table 2:** MIC values of compounds **1**–**6** in μg/mL against five bacterial strains.

Compounds	*Staphylococcus aureus*	*Bacillus thuringiensis*	*Bacillus subtilis*	*Escherichia coli*	*Shigella dysenteriae*
**1**	>200	>200	125	>200	>200
**2**	125	62.5	62.5	>200	>200
**3**	>200	125	125	>200	>200
**4**	>200	>200	>200	>200	>200
**5**	31.3	31.3	15.6	62.5	62.5
**6**	15.6	31.3	15.6	62.5	62.5
KS	3.9	3.9	3.9	3.9	3.9
C	>200	>200	>200	7.8	7.8

KS = Kanamycin sulfate, C = Cefradine.

**Table 3:** Cytotoxicity of compounds **1**–**6** (IC_50_, µM).

Compounds	A549	HeLa	HepG2
**1**–**4**	>100	>100	>100
**5**	32.37 ± 3.75	25.64 ± 2.34	41.87 ± 6.53
**6**	30.65 ± 3.87	18.36 ± 1.72	39.44 ± 3.61
Adriamycin	0.68 ± 0.06	0.46 ± 0.05	1.23 ± 0.02

Values represent mean ± SD (*n* = 3) based on three individual experiments.

## Supplementary Materials

The following are available online, HR-ESI-MS and NMR spectra data of compounds **1** and **2** as supporting information.
