# Supplementary figures and images for: Two New Thymol Derivatives from the Roots of Ageratina adenophora
Source: Molecules. 2017 Apr 8;22(4):592. doi: 10.3390/molecules22040592 (PMC6154539; doi:10.3390/molecules22040592)

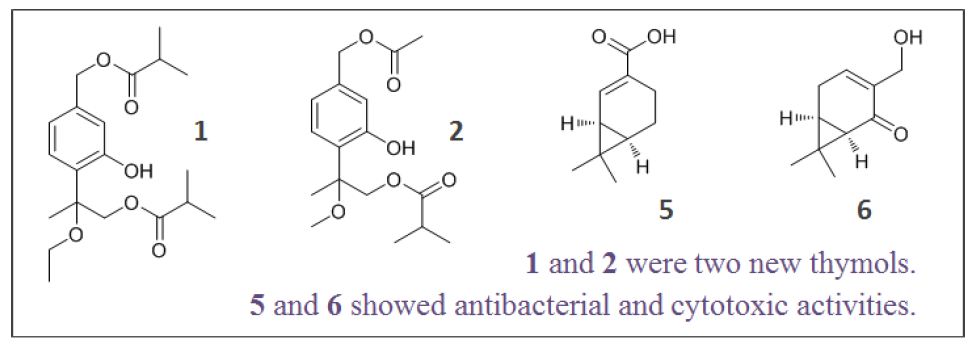

Supplement: Supplementary file 1 [file molecules-22-00592-s001.jpg]
